# Modified ERCP Technique for Choledocholithiasis in Situs Inversus Totalis

**DOI:** 10.14309/crj.0000000000002088

**Published:** 2026-04-24

**Authors:** Alya Binmahfouz

**Affiliations:** 1 Diagnostic Imaging Department, King Abdulaziz University Hospital, Jeddah, Saudi Arabia

**Keywords:** situs inversus totalis, ERCP, CBD stones

## Abstract

Situs inversus totalis (SIT) is a rare congenital condition characterized by a complete mirror-image reversal of the thoracic and abdominal organs. Although usually asymptomatic, SIT poses significant challenges during diagnostic and therapeutic procedures due to reversed anatomy. Endoscopic retrograde cholangiopancreatography (ERCP) is the standard treatment of biliary and pancreatic ductal diseases, yet it becomes technically demanding in patients with SIT. We report a case of choledocholithiasis in a patient with SIT successfully managed with ERCP, and briefly review relevant procedural considerations.

## INTRODUCTION

Situs inversus totalis (SIT) is a rare congenital condition characterized by a complete mirror-image reversal of the thoracic and abdominal organs. Although usually asymptomatic, SIT poses significant challenges during diagnostic and therapeutic procedures due to reversed anatomy. Endoscopic retrograde cholangiopancreatography (ERCP) is the standard treatment of biliary and pancreatic ductal diseases, yet it becomes technically demanding in patients with SIT. We report a case of choledocholithiasis in a patient with SIT successfully managed with ERCP, and briefly review relevant procedural considerations.^1–5^

## CASE REPORT

**Figure 1.**
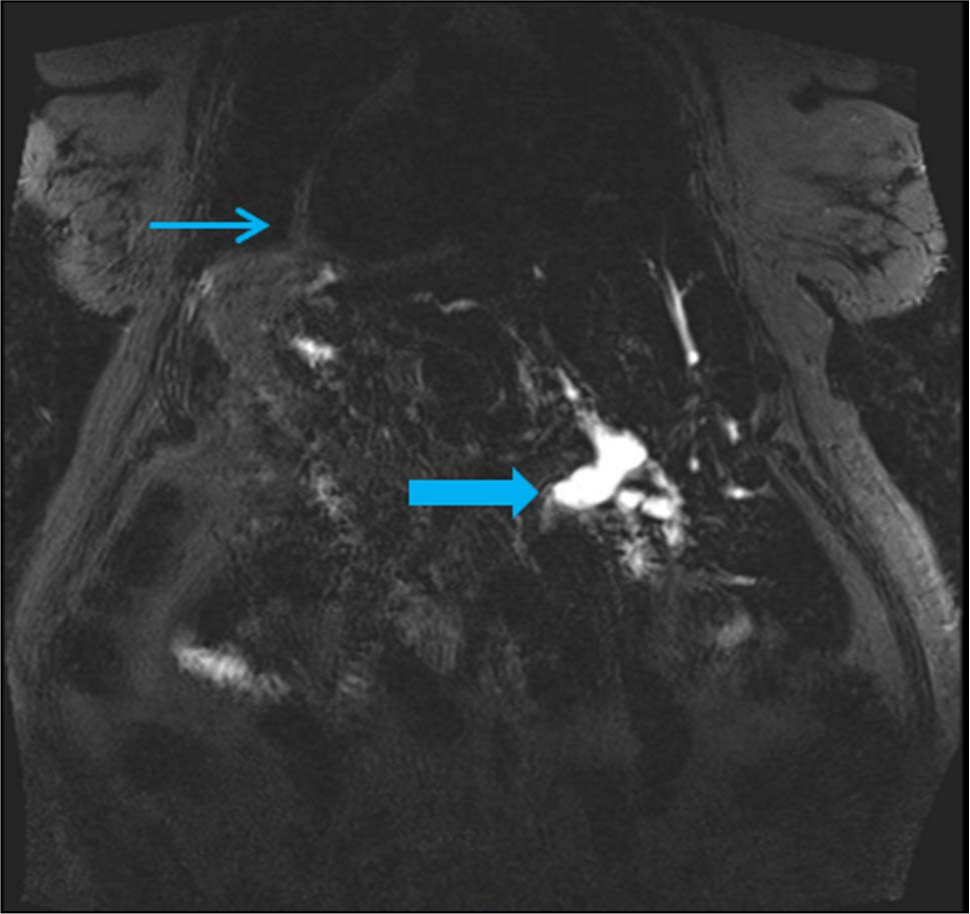
Coronal MRI demonstrates markedly dilated CBD with distal tapering and abrupt cutoff of the CBD with multiple filling defects (thick arrow). Situs inversus and dextrocardia (thin arrow) are seen. CBD, common bile duct; MRI, magnetic resonance imaging.

**Figure 2.**
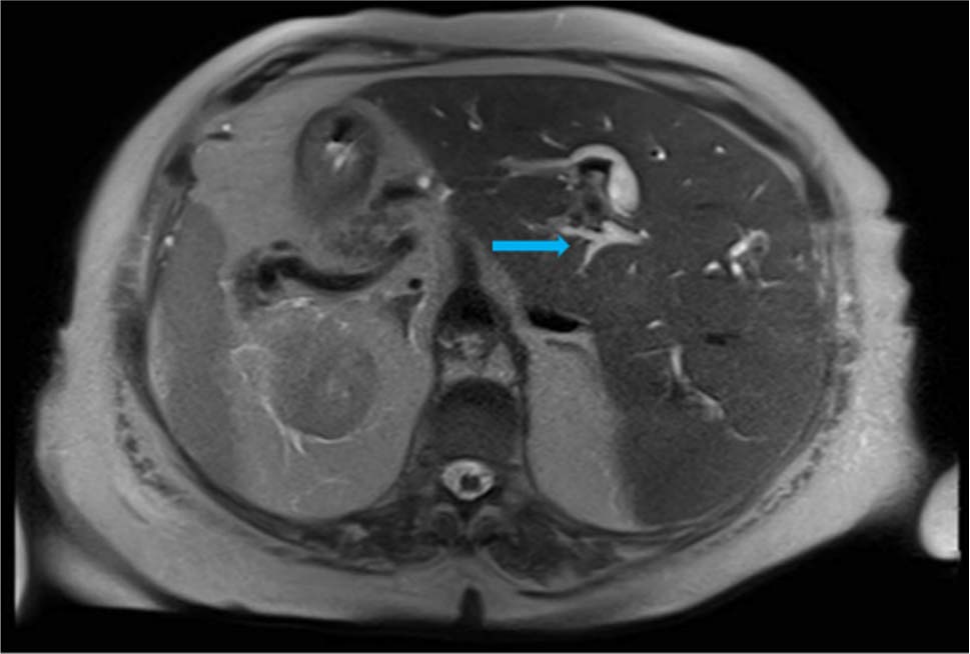
Axial magnetic resonance imaging demonstrates intrahepatic biliary tree dilatation (arrow). Transposition of the liver, spleen, and pancreas are noted.

A 70-year-old woman, known case of SIT with multiple comorbidities (coronary artery disease, hypertension, type 2 diabetes mellitus, and obesity) transferred from another hospital with fever, epigastric pain, and left upper quadrant tenderness. No jaundice was noted on physical examination. Her blood tests showed elevated liver enzymes, low protein and albumin levels, and normal total and direct bilirubin. Abdominal ultrasound performed at the referring hospital showed gallstones with multiple obstructive common bile duct (CBD) stones. Magnetic resonance imaging/magnetic resonance cholangiopancreatography (MRI/MRCP) showed multiple distal CBD filling defects with marked CBD dilatation and abrupt cutoff with distal tapering, associated with mild intra/extrahepatic biliary dilatation (Figures 1 and 2). Mirror image transposition of abdominal viscera and dextrocardia are noted. In addition, esophagogastroduodenoscopy (EGD) was performed and showed mirror image endoscopic anatomy but failed to localize the papilla due to the presence of a large periampullary diverticulum.

**Figure 3.**
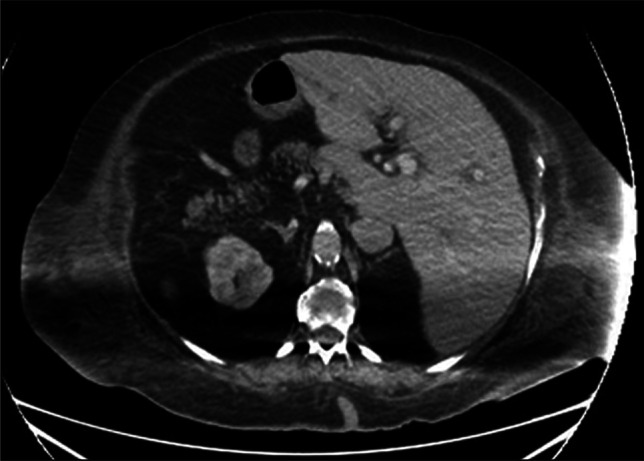
Axial computed tomography scan post endoscopic retrograde cholangiopancreatography demonstrates resolution of the dilated biliary system.

On admission, the patient was given intravenous tazocin, paracetamol, and ringer lactate. No endomethacin was administered. ERCP was initially performed in supine position based on anesthesia team preference; however, cannulation of the papilla was difficult, and the patient was turned into the right lateral position. Moreover, the anesthesiologist decided to put the patient under conscious sedation instead of general anesthesia or deep sedation to avoid postprocedural difficult extubation or ICU admission, which added more technical difficulty during the procedure. With endoscopist on her right side, successful intubation and cannulation of the inversed papilla at 2 o'clock position was achieved after 3 attempts using conventional single-wire technique. Incidental finding of a periampullary diverticulum prolonged the process (cannulation time about 7 minutes). Sphincterotomy was performed with good hemostasis, and balloon sweep retrieved multiple stones (the largest measured 8 mm) with complete clearance of CBD and free flow of bile noted endoscopically. No CBD stenting was required. The overall procedure lasted 40 minutes with no immediate postprocedural complications. The patient throughout the procedure was hemodynamically stable and maintained her oxygen saturation. She was discharged next day asymptomatic. Follow-up blood tests after 1 week showed normalization of liver enzymes and follow-up abdominal computed tomography showed patent CBD with improvement of biliary system dilatation (Figure 3).

## DISCUSSION

SIT is quite rare, with prevalence of 1 in 10,000–20,000 people worldwide. People with it have essentially mirror image of the major internal organs in the chest and abdomen. It is an autosomal recessive congenital condition and those people can live completely normal lives without any issues. It can be discovered incidentally during imaging or even during surgery, nonetheless, it can affect certain medical or surgical procedures just because doctors must keep the organ placement in mind. A well-known associated congenital anomaly is primary ciliary dyskinesia leading to Kartagener syndrome (situs inversus with chronic respiratory tract infections). In addition, congenital heart defects, great vessels malformations, polysplenia, and ectopic papilla have been reported.

Our case report represents an elderly lady with SIT and obstructive CBD stones necessitating ERCP with modified positioning and technique. She was transferred to our hospital after failed intervention at another center. Only a limited number of successful ERCPs in patients with SIT have been reported in the literature. Each case contributes valuable insights into technical modifications and endoscopic orientation strategies necessary for safe and effective intervention. Our patient is the second case reported in Saudi Arabia, along with a few case reports and case series from USA and China published in the past 5 years.

We emphasize the technical challenges of ERCP in SIT patients and the importance of experienced endoscopists capable of navigating the instruments in the opposite orientation to avoid potential complications like post ERCP pancreatitis, bleeding, perforation, or infection. Currently, there is no established consensus on the standard positioning for SIT patients undergoing ERCP. Patients could lie prone, supine, left lateral, right lateral, or even modify their position during the procedure at the discretion of the endoscopist. The endoscopist may even opt to use double-wire technique or transpancreatic precut to rescue inadvertent pancreatic duct cannulation.

We also highlight the crucial role of pre intervention imaging to address any associated congenital anomalies that may hinder the procedure, and to precisely plan the technique beforehand, raising its success rate. Typically, MRCP is considered the best noninvasive imaging modality for evaluation of the biliary and pancreatic ducts. It provides high resolution, detailed images without the risks associated with contrast agents. It is especially valuable for identifying the cause and level of biliary obstruction, detecting choledocholithiasis, and evaluating strictures or malignancies. Compared with computed tomography and ultrasound, MRCP offers superior soft tissue contrast and ductal visualization, making it the preferred diagnostic step before ERCP, which is now reserved primarily for therapeutic interventions rather than diagnosis. With careful technique and preparation, ERCP can be safely performed in these patients.

## DISCLOSURES

Author contributions: A. Binmahfouz collected the data and images of this patient, wrote the manuscript, and reviewed the literature.

Financial disclosure: None to report.

Informed consent was obtained for this case report.

## ABBREVIATIONS:

CBD: common bile duct
EGD: esophagogastroduodenoscopy
ERCP: endoscopic retrograde cholangiopancreatography
ICU: intensive care unit
MRI/MRCP: magnetic resonance imaging/magnetic resonance cholangiopancreatography
SIT: situs inversus totalis
